# Factors Associated With Older Adults’ In-Hospital Mobility: A Comparison Between Israel and Denmark

**DOI:** 10.1093/geroni/igab046.093

**Published:** 2021-12-17

**Authors:** Anna Zisberg, Janne Petersen, Ove Andersen, Efrat Shadmi, Ksenya Shulyaev, Efrat Gil, Maayan Agmon, Mette Merete Pedersen

**Affiliations:** 1 University of Haifa, Haifa, HaZafon, Israel; 2 Copenhagen University Hospital Frederiksberg, Frederiksberg, Hovedstaden, Denmark; 3 Copenhagen University Hospital Hvidovre, Hvidovre, Hovedstaden, Denmark; 4 University of Haifa, Mount Carmel, Israel, Haifa, Hefa, Israel; 5 Faculty of Social Welfare and Health Science, University of Haifa, Haifa, Hefa, Israel; 6 Clalit health services, Haifa and West Galilee, Israel, Haifa, HaZafon, Israel; 7 The Cheryl Spencer Department of Nursing, University of Haifa, Israel, Haifa, HaZafon, Israel; 8 Copenhagen University Hospital, Amager and Hvidovre, Hvidovre, Hovedstaden, Denmark

## Abstract

Low levels of in-hospital mobility and excessive bed rest are widely described across the globe as a major risk factor for hospital associated disabilities. Different predictors of in-hospital and post-discharge mobility limitations have been proposed across studies, including age, admission diagnosis, physical performance, cognitive impairment, performance of activities of daily living, and length of stay. However, it is unknown whether similar risk factors across countries are associated with in-hospital mobility given different mobility measurement methods, variations in measurement of predictors and differences in populations studied. In the current study, we investigated the relationship between in-hospital mobility and a set of similar risk factors in functionally independent older adults (65+) hospitalized in acute care settings in Israel (N=206) and Denmark (N=113). In Israel, mobility was measured via ActiGraph and in Denmark by ActivPal for up to seven hospital days. Parallel analysis of covariance (ANCOVA) in each sample showed that community-mobility before hospitalization, mobility performance at admission and length of stay were associated with in-hospital mobility in both countries, whereas age and self-reported health status were associated with mobility only in Denmark. This comparison indicates that despite slightly different measurement approaches, similar risks are attributed to older adults’ low in-hospital mobility and emphasizes the contribution of commonly used pre-hospitalization mobility measures as strong and consistent risk factors. This knowledge can support a better understanding of the need of both standard risk assessments and country-based tailored approaches.

